# Mini-Review: Mixed Ionic–Electronic Charge Carrier Localization and Transport in Hybrid Organic–Inorganic Nanomaterials

**DOI:** 10.3389/fchem.2020.00537

**Published:** 2020-07-14

**Authors:** Mariano Romero, Dominique Mombrú, Fernando Pignanelli, Ricardo Faccio, Alvaro W. Mombrú

**Affiliations:** Centro NanoMat & Área Física, Departamento de Experimentación y Teoría de la Estructura de la Materia y sus Aplicaciones - DETEMA, Facultad de Química, Universidad de la República, Montevideo, Uruguay

**Keywords:** hybrid organic–inorganic composites, nanomaterials, mixed ionic–electronic conducting materials, semiconductor, Raman micro spectroscopy, impedance spectroscopy

## Abstract

In this mini-review, a comprehensive discussion on the state of the art of hybrid organic–inorganic mixed ionic–electronic conductors (*hOI*-MIECs) is given, focusing on conducting polymer nanocomposites comprising inorganic nanoparticles ranging from ceramic-in-polymer to polymer-in-ceramic concentration regimes. First, a brief discussion on fundamental aspects of mixed ionic–electronic transport phenomena considering the charge carrier transport at bulk regions together with the effect of the organic–inorganic interphase of hybrid nanocomposites is presented. We also make a recount of updated instrumentation techniques to characterize structure, microstructure, chemical composition, and mixed ionic–electronic transport with special focus on those relevant for *hOI*-MIECs. Raman imaging and impedance spectroscopy instrumentation techniques are particularly discussed as relatively simple and versatile tools to study the charge carrier localization and transport at different regions of *hOI*-MIECs including both bulk and interphase regions to shed some light on the mixed ionic–electronic transport mechanism. In addition, we will also refer to different device assembly configurations and *in situ/operando* measurements experiments to analyze mixed ionic–electronic conduction phenomena for different specific applications. Finally, we will also review the broad range of promising applications of *hOI*-MIECs, mainly in the field of energy storage and conversion, but also in the emerging field of electronics and bioelectronics.

## Introduction

In the last decades, mixed ionic–electronic conductors (MIECs) have been widely studied for energy storage and energy conversion materials, separation membranes, and catalysts (Shao and Haile, 2004; Maier, 2005; Wachsman and Lee, 2011; Aoki et al., 2014). Both ionic (σ_*i*_) or electronic (σ_*e*_) conduction obey separately and analogously to the following equation:

(1) $$\begin{array}{r} \sigma =qN\mu \end{array}$$

where *q* is the charge, *N* is the number, and μ is the mobility of the charge carrier, the latter being proportional to diffusivity (*D*). In the particular case of inorganic MIECs, some well-known examples are semiconducting compounds such as Ag_2_X (with *X* = S, Se, or Te) as mixed silver ion (Ag^+^) and electronic conducting materials (Yokota, 1961; Miyatani, 1973; Riess, 2003) and A-doped MO_2−δ_ (typically *M* = Ce or Zr, and A being different dopants) as mixed oxygen ion (O^2−^) and electronic transport materials (Goodenough, 2000; Balaguer et al., 2011; Lin et al., 2015). However, one of the most relevant inorganic MIEC materials gaining special attention in the recent years are A_X_M_2_O_4_ (with *M* = Ni, Co, and/or Mn and *A* = Li or Na) due to their excellent performance, particularly as cathode materials for lithium (Li^+^) and sodium (Na^+^) ion batteries (Doeff et al., 1993; Barker et al., 1996; Saïdi et al., 1996; Thackeray, 1997; Dokko et al., 2001; Lu and Dahn, 2001; Cao and Prakash, 2002; Levasseur et al., 2002; Sauvage et al., 2007; Berthelot et al., 2010; Tevar and Whitacre, 2010). For instance, typical electronic conductivities (σ_*e*_) and lithium-ion diffusivities (*D*_*i*_) for Li_X_M_2_O_4_ cathode materials are σ_*e*_ ~ 10^−6^-10^−1^ S cm^−1^ and *D*_*i*_ ~ 10^−11^-10^−8^ cm^2^s^−1^, respectively, depending strongly on the transition metal (M), lithiation degree (*x*), and crystallinity (Park et al., 2010). In the particular case of semiconducting inorganic nanomaterials, both ionic and electronic transport present lower charge carrier resistance at the crystalline bulk regions but are drastically compromised by the poor charge carrier conducting nature of grain boundaries (Park et al., 2010). In the last decades, the addition of conducting coating materials and secondary phases such as mixed ionic–electronic conducting organic materials (e.g., conducting polymers), working as linkers between inorganic nanomaterials, has attracted a lot of attention (Judeinstein and Sanchez, 1996; Gómez-Romero and Lira-Cantú, 1997; Guizard et al., 2001; Le Bideau et al., 2011). It is well-accepted that electronic conducting organic polymers, usually called conjugated polymers, are semiconductors in nature and that the most popular cases such as poly(pyrrole) (Ppy) (Della Santa et al., 1997), poly(aniline) (PANI) (Zhang K. et al., 2012a; Chatterjee et al., 2013; Zhang Q. et al., 2013a; Roussel et al., 2015), poly(ethylenedioxythiophene) (PEDOT) (Crispin et al., 2006; Udo et al., 2009; Takano et al., 2012; Kim et al., 2013; Mengistie et al., 2013, 2015; Lee et al., 2014; Kumar et al., 2016; Zia Ullah et al., 2016), and poly(3-hexylthiophene) (P3HT) (Zhang Q. et al., 2012; Pingel and Neher, 2013; Glaudell et al., 2015; Jacobs et al., 2016; Qu et al., 2016; Jung et al., 2017; Wang W. et al., 2017; Lim et al., 2018) generally exhibit an electronic donor behavior. In this case, the most common procedure to enhance the electronic conduction, where charge carriers will be mostly holes rather than electrons, is by doping these polymers with electronic acceptor species (p-type doping) such as halide and sulfonate salts, yielding a decrease in the electronic band gap and an increase of the electronic conductivity up to σ_*e*_ ~ 10^−1^-10^3^ S cm^−1^ values (Della Santa et al., 1997; Crispin et al., 2006; Udo et al., 2009; Takano et al., 2012; Zhang K. et al., 2012; Zhang Q. et al., 2012, 2013; Chatterjee et al., 2013; Kim et al., 2013; Mengistie et al., 2013, 2015; Pingel and Neher, 2013; Lee et al., 2014; Glaudell et al., 2015; Roussel et al., 2015; Jacobs et al., 2016; Kumar et al., 2016; Qu et al., 2016; Zia Ullah et al., 2016; Jung et al., 2017; Wang W. et al., 2017; Lim et al., 2018). The mere presence of the dopant, typically halide, or sulfonate salts with relatively high degree of dissociation, will trigger a non-negligible ionic conduction in addition to the electronic transport (Riess, 2000). It is important to mention that there are other “non-dissociable” excellent dopants such as the case of tetracyanoquinodimethane (TCNQ) in all of its fluorinated forms, but as it does not provide highly mobile ionic carriers, it will not be considered in this review. It was long observed that protons (H^+^), lithium (Li^+^), sodium (Na^+^), or potassium (K^+^) cations yielded a considerable ionic contribution to the total mixed ionic–electronic transport of conjugated polymers (Nigrey et al., 1978; Aldebert et al., 1986; Barthet and Guglielmi, 1995; Watanabe, 1996). The voluminous dopant anions are generally more fixed to the polymer chain, allowing the electronic exchange process (doping) to take place but contributing in a lesser extent to the ionic conductivity except for a few particular cases (Cheng et al., 2005). Pursuing an increase in the ionic conduction of MIECs, blending and co-polymerization (including functionalization of side chains) of electronic conducting polymers with good ionic conducting polymers [e.g., poly(ethylene oxide) (PEO)], has shown enhancement of ionic conductivities up to σ_*i*_ ~ 10^−5^-10^−4^ S cm^−1^ (Li and Khan, 1991; Barthet et al., 1997; Ghosh and Inganäs, 2000; Zhang et al., 2002; Patel et al., 2012; Ju et al., 2014; Kang et al., 2014; Dong et al., 2019; Sengwa and Dhatarwal, 2020). Another strategy includes the simultaneous doping and blending of electronic conducting polymers with polymeric dopants, particularly observed for protons and lithium-ion charge carriers (Murthy and Manthiram, 2011; Fu and Manthiram, 2012; Liu et al., 2012). However, it is important to remark that the inclusion of electronic-insulating polymers inevitably leads to the declining of the electronic conductivity (σ_*e*_ ~ 10^−5^ S cm^−1^, i.e., several orders of magnitude less than the isolated conducting polymer in its doped form), and thus, electronic-conducting polymer/ionic-conducting polymer/dopant concentrations need to be rationally balanced (Li and Khan, 1991; Barthet et al., 1997; Ghosh and Inganäs, 2000; Zhang et al., 2002; Murthy and Manthiram, 2011; Fu and Manthiram, 2012; Liu et al., 2012; Patel et al., 2012; Ju et al., 2014; Kang et al., 2014; Dong et al., 2019; Sengwa and Dhatarwal, 2020). Recent comprehensive reviews discussing different types of organic MIEC classes, with particular focus on taxonomy and electronic–ionic interactions, are given by Paulsen et al. (2020), and a thorough discussion of morphologic effects on organic polymeric MIEC is given by Onorato and Luscombe (2019). On the other hand, it is well-known that the addition of semiconducting ceramic nanoparticles, even with negligible intrinsic electronic (or ionic) transport ability, can also yield an enhancement of the electronic (or ionic) conduction in conducting polymer nanocomposites. For instance, the presence of inorganic nanoparticles, particularly transition metal oxides, has yielded a notorious increment of electronic conductivity for electronic–conductor polymer nanocomposites in both ceramic-in-polymer (Mombrú et al., 2017a,b; Mombrú et al., 2019) and polymer-in-ceramic concentration regimes (Huguenin et al., 2004; Wang et al., 2010; Mombrú et al., 2017a). In analogy, the presence of inorganic nanoparticles resulted in an enhancement on the ionic conductivity for ionic conductor polymer nanocomposites (Kloster et al., 1996; Scrosati et al., 2000; Shin and Passerini, 2004). The presence of secondary phases or inorganic nanofillers induces slight structural modifications, altering the degree of order of the conducting polymer chains that could explain the enhancement of the conductivity, without considering direct mediation of charge carriers through the nanoparticle interphase. Although it is accepted that the electronic conduction in polymer nanocomposites is usually related to higher crystallinity (or higher degree of order), the enhancement of the ionic conduction is mostly associated to lower crystallinity (or lower degree of order), but the latter case is still under recent debate (Onorato and Luscombe, 2019). Furthermore, in the case of ceramic nanoparticles' interaction with conducting polymers, the presence of an interphase between both organic and inorganic materials adds a particular complexity to the system and can eventually lead to important consequences in both ionic and electronic transport properties. Leaving out drastic effects such as voids, poor contact, or the presence of decomposition phases due to eventual chemical reactions, it is extremely difficult to obtain well-defined interphases between such different materials. For instance, the presence of defects, mainly in the inorganic nanoparticle boundaries, can lead to the presence of charge localization at the interphase and the presence of different crystallographic surfaces of the inorganic nanoparticle at the interphase can exhibit different electronic interactions with the polymer phase. Up to now, to the best of our knowledge, there are only a few reviews of MIEC materials with particular focus on their applications such as energy (Sengodu and Deshmukh, 2015), bioelectronics (Han S. et al., 2019), and sensing (Inal et al., 2018), but no further insights into *hOI*-MIECs. In this mini-review, charge carrier localization and transport at different regions of *hOI*-MIECs including both bulk and interphase regions is revised, focusing on the use of some powerful and versatile instrumental techniques.

## Charge Carrier Localization

**Figure 1:**
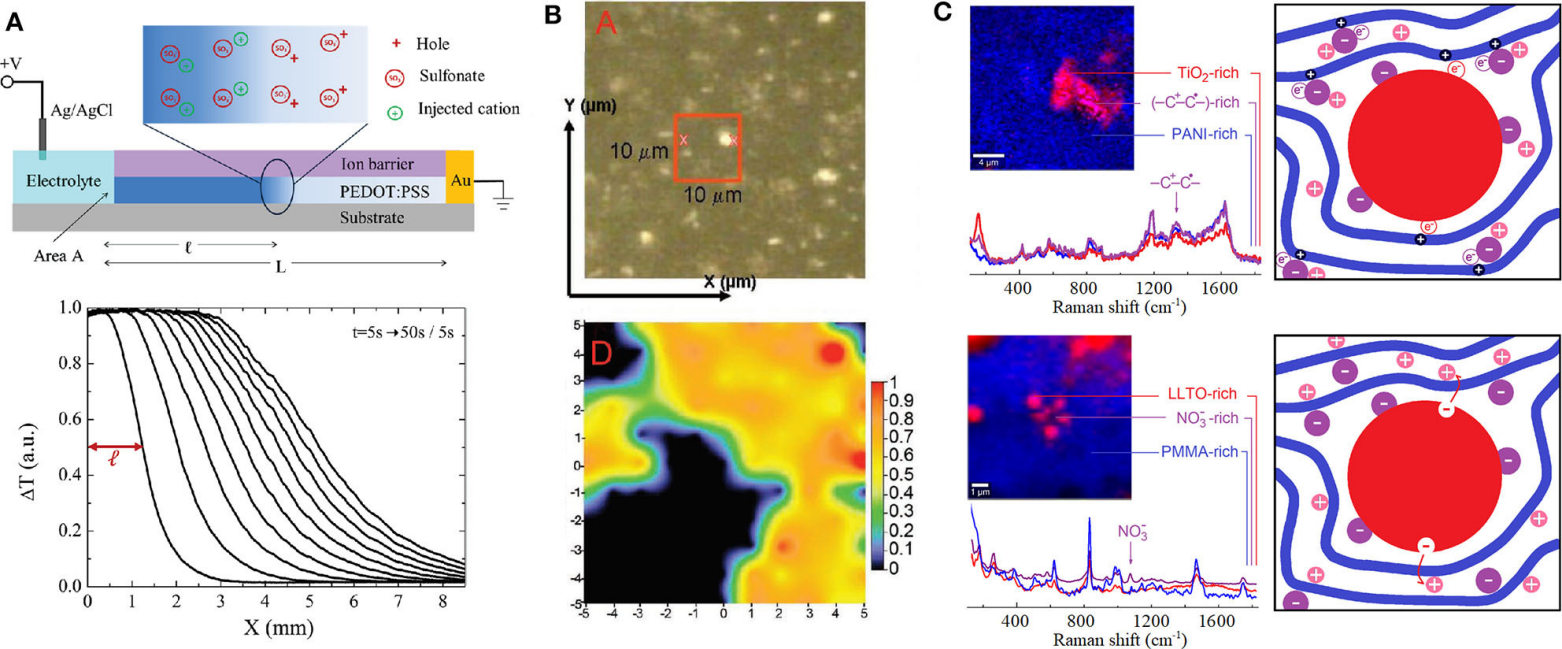
**(A)** Schematization indicating the charge distribution around the dedoping front (upper panel) and evolution of dedoping front where Δ*T* is the change of transmitted light intensity with respect to the zero bias state during the injection of potassium cations for PEDOT:PSS film (lower panel) (Stavrinidou et al., 2013). This figure was used and adapted/altered minimally with permission from John Wiley and Sons. **(B)** Optical image (upper panel) and Raman imaging (lower panel) providing a semi-quantitative measure of the Li_1−x_(Ni_y_Co_z_Al_1−y−z_)O_2_ (NCA) cathode state of charge (SOC) where the dark region is associated to carbon-rich zone and the colored region is associated to the NCA-rich zone ranging from blue (lower SOC) to red (higher SOC) (Nanda et al., 2011). This figure was used and adapted/altered minimally with permission from John Wiley and Sons. **(C)** Raman imaging and schematization of charge carrier localization near hybrid organic–inorganic interphases for electronic conducting polymer nanocomposite (sulfonic acid-doped polyaniline with embedded TiO_2_ nanoparticles; Mombrú et al., 2017a) (upper panel) and ionic conducting polymer nanocomposite (lithium nitrate solid polymethylmethacrylate electrolyte with embedded Li_0.3_La_0.7_TiO_3_ nanoparticles; Romero et al., 2016) (lower panel). References for schematization are as follows: organic polymer (blue), inorganic nanoparticles (red), dopant cation (+, in pink), dopant anion (–, in purple), and electronic charge carriers (+, in dark blue). Micro-Raman images and spectra are portions of figures adapted/altered minimally with permission from Elsevier.

There are a lot of instrumentation techniques that can provide particularly rich information about structural features of *hOI*-MIECs such as Nuclear Magnetic Resonance (NMR), X-ray diffraction (XRD), and wide-/small-angle X-ray scattering (WAXS/SAXS) in both transmission or grazing incidence configurations (Sanjeeva Murthy, 2016). However, it is important to remark that X-ray scattering techniques are relatively accessible but generally give indirect information about charge carrier localization and on the other hand, although NMR could be very powerful to monitor charge carrier's location, it is particularly less versatile than other optical spectroscopies techniques. For instance, a relatively simple and powerful method to monitor not only charge localization but also drift mobility in organic MIECs is the “moving front” experiment, which is based on visible light transmission monitoring through an electrochromic film as it is dedoped due to lateral injection of H^+^, Na^+^, or K^+^ ions from a planar junction with an electrolyte, as shown in Figure 1A (Stavrinidou et al., 2013; Rivnay et al., 2016). Nonetheless, one of the most popular but no less powerful and versatile technique to study structural features of *hOI*-MIECs is vibrational spectroscopy. Raman spectroscopy is particularly interesting for inorganic materials characterization as it does not exclude highly amorphous systems in comparison with XRD and provides accessibility to vibrational modes with lower wavenumbers (typically ν_min_ ~ 80–100 cm^−1^) in comparison to infrared spectroscopy (typically ν_min_ ~ 200–400 cm^−1^). Raman spectroscopy also has the remarkable advantage of needing little sample preparation, allowing the study of materials in its native conditions, as well as permitting collection of *in situ* and *in operando* measurements. For instance, *in situ*/*operando* Raman spectroscopy has allowed the study of the state of charge of (Li, Na, K)_X_M_2_O_4_ electrodes by monitoring the broadening and shifting of Raman peaks when lowering Li, Na, or K content from nominal *X* = 1 (full charged cathode), particularly associated to the loss of ions from the interlayer of the MO_2_ layered structure (Dokko et al., 2003; Nanda et al., 2011; Nishi et al., 2013; Chen et al., 2015; Flores et al., 2018). An example on the use of Raman imaging to monitor the state of charge for a Li_1−x_(Ni_y_Co_z_Al_1−y−z_)O_2_ cathode is shown and described briefly in Figure 1B (Nanda et al., 2011). In addition, the use of micro-Raman imaging technique is highly powerful to study simultaneously both compositional and microstructural features, especially for hybrid inorganic–organic materials, as the characteristic Raman signals for inorganic and organic compounds generally lie well-separated at lower (ν < 800 cm^−1^) and higher (ν > 800 cm^−1^) wavenumbers, respectively (Romero et al., 2016; Mombrú et al., 2017a,b,c; Pignanelli et al., 2018, 2019a,b). Furthermore, although Raman spectroscopy is quite sensitive to diluted effects such as doping processes of inorganic materials, it is on the other hand, extremely sensitive to doping effects of organic materials such as conducting polymers (Furukawa, 1996). Briefly, the doping process of conducting polymers yields to drastic modifications of the Raman signature in relation to the charge carrier formation, typically in the form of positive polarons (–C^+^-C^•^-) or bipolarons (–C^+^-C^+^-), particularly altering both Raman frequency and activity of vibrational modes associated to carbon-to-carbon (C=C) molecular bonds in conjugated polymers (Furukawa, 1996; Kumar et al., 2012; Yamamoto and Furukawa, 2015; Francis et al., 2017; Mombrú et al., 2018; Nightingale et al., 2018). For instance, micro-Raman imaging has evidenced the presence of these types of charge carriers particularly localized near the interphase with inorganic nanoparticles; [e.g., MX_2_ with M being different transition metals and X = O (for oxides) or *S* (for sulfides) (Mombrú et al., 2017a,b,c; Mombrú et al., 2019)]. The increment of conducting polymer electronic charge carriers near the interphase could be discussed in view of at least two eventual scenarios: (one or *passive*) the dopant stabilizes at the interphase due to strong polar or coulombic interactions with nanoparticles surface, or/and (two or *active*) the nanoparticles are also good electronic acceptors, producing in both cases an enhancement on the doping of nearby polymer chains, as schematized in Figure 1C (upper panel). On the other hand, micro-Raman imaging has also been useful to evidence the enhancement of ionic-pair dissociation occurring near the interphase with inorganic nanoparticles, in agreement with the increment of ionic conductivity (Romero et al., 2016; Pignanelli et al., 2018, Pignanelli et al., 2019a). Analogously, two different scenarios could be discussed for ionic charge carriers: (one or *passive*) the counter-ion (in analogy to the dopant anion) stabilizes at the interphase due to strong polar or coulombic interactions with nanoparticles surface yielding an enhancement on the ionic-pair dissociation, or/and (two or *active*) the nanoparticles may also possess mobile ionic carriers at the surface (e.g., active filler) that can be injected into the polymer, as schematized in Figure 1C (lower panel). Whatever the case, the previous micro-Raman imaging studies revealed that the interphase of organic–inorganic nanocomposites, to a greater or lesser extent, always play an important role in the charge carrier transport mechanism.

## Charge Carrier Conduction

**Figure 2:**
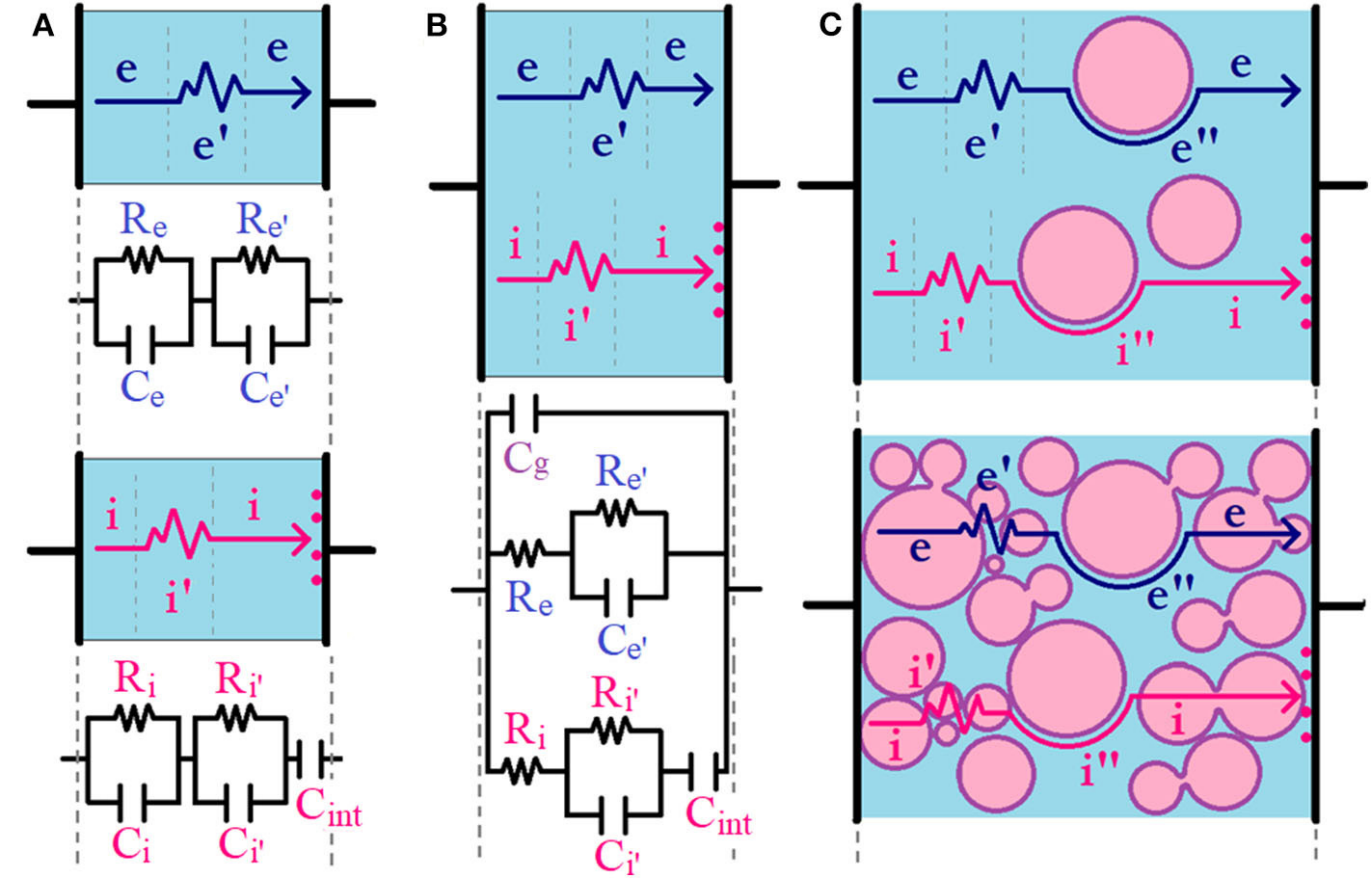
Circuit model schematization for **(A)** separated electronic and ionic transport in a single phase, **(B)** mixed ionic–electronic transport in a single phase, and **(C)** mixed ionic–electronic transport in *hOI*-MIECs ranging from ceramic-in-polymer (upper panel) to polymer-in-ceramic (lower panel). Electronic and ionic hypothetical pathways are shown with dark blue and pink arrows. The zigzag part of the arrows indicates the presence of eventual grain boundaries or depleted regions [with associated ionic (*i*′) or electronic (*e*′) contributions] and the curved part of the arrows indicates the presence of eventual transport pathway mediated through organic–inorganic interfacial regions [with associated ionic (*i*″) and electronic (*e*″) contributions].

There are several electrochemical methodologies to study the charge carrier conduction in MIECs, but one of the most powerful techniques to access both electronic and ionic transport simultaneously is impedance spectroscopy (Jamnik and Maier, 1999; Vorotyntsev et al., 1999; Huggins, 2002; Atkinson et al., 2004; Lee et al., 2009). Briefly, the impedance response as a function of the frequency (typically 10^−3^-10^6^ Hz) of an oscillating voltage (typically 10–100 mV amplitude) can provide information about different charge carriers with different relaxation times (τ) depending on their *q*/*m* ratio; [i.e., the higher the *q*/*m* ratio, the lower τ and the higher associated frequencies (*f* = 2π/τ)]. In this case, the Nyquist representation of impedance (imaginary impedance vs. real impedance, –*Z*″ vs. *Z*′) for a single electronic semiconductor in a continuous medium will show a single semicircle arc. The semicircle arc associated to the electronic carrier transport can be typically modeled using the parallel combination of a resistor (*R*_*e*_) and a capacitor (*C*_*e*_). In analogy, but with probably higher associated τ (lower *f*), a single ionic conductor in a continuous medium will also show a similar single semicircle arc associated to the ionic carrier transport that can also be modeled using the parallel combination of a resistor (*R*_*i*_) and a capacitor (*C*_*i*_), whose associated charge carrier pathway is represented with a straight line in Figure 2A. If an additional pathway is mediating the electronic (or ionic) transport (e.g., the presence of grain boundaries or depletion regions in less crystalline solids), a second $R_{e^{\prime }}$$C_{e^{\prime }}$ (or $R_{i^{\prime }}$$C_{i^{\prime }}$) parallel combination connected in series with the previous one is usually necessary to fit the total impedance response, whose associated charge carrier pathway is represented with a zig-zag line in Figure 2A. For simplicity, from now on, we will only consider the charge carrier transport of ionic and electronic conductor samples assembled in a symmetric cell configuration using ideal metallic ion-blocking electrodes. This means that only electronic carriers will be short-circuited and ionic species will be blocked at the interphase with the ion-blocking metallic electrodes but the opposite will apply in the case of using electronic-blocking electrodes. In the case of using metallic ion-blocking electrodes, in addition to the semicircle arc observed at higher frequencies, the Nyquist plots of single ionic conductors will also show an additional capacitive tail at low frequencies (*C*_*int*_), which is associated to the polarization of blocked ions at the sample/electrode interphase, as shown in Figure 2A. If now we consider the simplest case of a MIEC material, the bi-continuous ionic and electronic channels can be strategically represented by the parallel combination of ionic and electronic resistances (*R*_*i*_ and *R*_*e*_, respectively) together with a global geometrical capacitance (*C*_*g*_), with the associated pathway represented by a straight line in Figure 2B. It is important to remark that the *C*_*int*_ element only appears connected in series with the ionic resistance as we are working with ideal ion-blocking electrodes, but the opposite will occur (i.e., an analogous *C*_*int*_ element will only appear connected in series with the electronic resistance) if we are working with electronic-blocking electrodes. The origin of this circuit model simplification is described thoroughly by Jamnik and Maier and is only applicable for macroscopically thick samples considering ideal selectively ion-blocking electrodes and chemical capacitance much larger than the interfacial capacitance of the blocked carriers (Jamnik and Maier, 1999; Lee et al., 2009). In the case that any of the electronic or ionic transport is mediated by the presence of a secondary pathway in a MIEC, generally associated to grain boundaries or depleted regions, as we discuss before, a second $R_{e^{\prime }}$$C_{e^{\prime }}$ (or $R_{i^{\prime }}$$C_{i^{\prime }}$) parallel combination connected in series with *R*_*e*_ (or *R*_*i*_), respectively, could be useful to fit the total impedance response, with associated pathway represented by a zig-zag line in Figure 2B (Huggins, 2002). In the recent literature, both the inclusion and exclusion of this second $R_{e^{\prime }}$$C_{e^{\prime }}$ (or $R_{i^{\prime }}$$C_{i^{\prime }}$) parallel combination in biphasic polymeric MIECs have been observed, depending mainly on the electronic- and ionic-conducting phase concentration or microstructural differences (Patel et al., 2012; Renna et al., 2017). In the particular case of *hOI*-MIECs, the second contribution (and probably a third contribution) to ionic or electronic transport could be present due to the mere existence of the organic–inorganic interphase, as shown in Figure 2C. However, even for a simplified experiment configuration, (e.g., using symmetric ion-blocking electrodes), it is important to rationalize the number of elements in a given circuit model to avoid over-parametrization. For instance, in the extreme case of *hOI*-MIECs based on a continuous organic semiconductor, [e.g., conducting polymer with diluted inorganic nanoparticle additives (ceramic-in-polymer)], both electronic and ionic carriers will be mainly transported through the organic matrix. For instance, $R_{e^{\prime }}$$C_{e^{\prime }}$ and $R_{i^{\prime }}$$C_{i^{\prime }}$ elements could be eventually excluded from the circuit model in the presence of homogeneous (full crystalline or amorphous) polymeric phase. However, in consonance with the non-homogeneous localization of charge carriers discussed in the previous section, the presence of an organic–inorganic interphase can eventually activate another electronic or/and ionic pathway mediated through the interphase that could be passive or active (Irvine et al., 1990). For instance, solid polymer electrolytes with active inorganic nanofillers are the typical case of organic–inorganic interphase-mediated ionic transport (Zheng et al., 2016; Yang et al., 2017; Pignanelli et al., 2019a), and a similar behavior will be observed for the electronic counterpart, if there are electronic interactions at the organic–inorganic interphase (Chen et al., 2010; Nowy et al., 2010; Cai et al., 2012; Mombrú et al., 2017b). This effect, whose associated charge carrier pathway is represented by a curved line in Figure 2C, can also be eventually modeled with $R_{e^{\prime \prime }}$$C_{e^{\prime \prime }}$ (or $R_{i^{\prime \prime }}$$C_{i^{\prime \prime }}$) elements connected in series with the electronic (or ionic) part of the mixed ionic–electronic circuit, in analogy to $R_{e^{\prime }}$$C_{e^{\prime }}$ (or $R_{i^{\prime }}$$C_{i^{\prime }}$), respectively. However, as mentioned earlier in the previous section, even when the inorganic nanoparticles are passive or non-interacting in nature with charge carriers, the concentration of both electronic or ionic charge carriers at the vicinities of the organic–inorganic interphase could also be activating a second pathway to the charge carrier transport. Nonetheless, in the case of passive interphases, this effect could be rather weak and both charge carrier transport pathways are expected to be mainly through the organic phase without interphase mediation; thus, only a global contribution to the charge carrier transport is usually observed and additional $R_{e^{\prime \prime }}$$C_{e^{\prime \prime }}$ (or $R_{i^{\prime \prime }}$$C_{i^{\prime \prime }}$) elements are not necessary to fit the global impedance response. In the other extreme case, [i.e., *hOI*-MIECs based on inorganic semiconductor nanoparticles with diluted organic polymeric additives (polymer-in-ceramic)], both electronic and ionic carriers are mainly transported through the inorganic matrix. In this case, due to the inevitable presence of grain boundaries in inorganic semiconductor nanoparticles, $R_{e^{\prime }}$$C_{e^{\prime }}$ (or $R_{i^{\prime }}$$C_{i^{\prime }}$) elements should always be considered, as this contribution practically governs the global electronic (or ionic) transport. In this case, the polymeric additions usually act as fillers of empty spaces between nanoparticles, resulting in an enhancement of the electronic (or ionic) conductivity, and this is usually evaluated directly on $R_{e^{\prime }}$$C_{e^{\prime }}$ (or $R_{i^{\prime }}$$C_{i^{\prime }}$) elements. However, in the case of simultaneous presence of particle-to-particle and particle–polymer–particle interphases, there will be at least two different pathways to electronic (or ionic) transport and additional $R_{e^{\prime \prime }}$$C_{e^{\prime \prime }}$ (or $R_{i^{\prime \prime }}$$C_{i^{\prime \prime }}$) elements could be necessary to fit the polymer-mediated transport contribution, as depicted in Figure 2C.

## Applications

The successful synergistic properties between organic and inorganic MIECs have yielded excellent performances, especially in the field of energy storage and particularly for lithium- and sodium-ion battery electrode materials (Sengodu and Deshmukh, 2015). In this sense, active cathode or anode materials embedded in polymeric hosts not only increase the mixed ionic–electronic conduction but also act as a sort of protection to the decomposition of active materials (Sengodu and Deshmukh, 2015). For instance, in the case of lithium-ion battery cathode materials: hybrid P3HT-co-PEO/LiFePO_4_ has improved the delivery of both ionic and electronic charge to active centers (Javier et al., 2011); Ppy/LiFePO_4_ with different hierarchical structures promoted both electronic and ionic transport (Fedorkova et al., 2010; Shi et al., 2017); PEDOT/LiFePO_4_ offers excellent discharge capacity (Vadivel Murugan et al., 2008); Ppy/α-LiFeO_2_ has improved the reversible capacity and cycling stability (Zhang et al., 2013); PPy/MoO_3_, PPy/V_2_O_5_, PPy/LiCoO_2_, and PPy/LiV_3_O_8_ yielded a reduction of charge transfer resistance of the Li^+^ ion intercalation/deintercalation process (Wang et al., 2010; Tian et al., 2011; Tang et al., 2012a,b; Liu et al., 2013); and PEDOT-co-PEG/LiNi_0.6_Co_0.2_Mn_0.2_O_2_ showed high discharge capacity and enhanced transport of Li^+^ ions as well as electrons (Ju et al., 2014). Furthermore, in the case of lithium-ion anode materials, only to mention some examples, hybrid Ppy/SnO_2_ yielded a more controlled Li^+^ diffusion (Yuan et al., 2007; Cui et al., 2011) and hybrid PANI-graphene/TiO_2_ yielded fast charge-to-discharge rate and high enhanced cycling performance (Zhang F. et al., 2012). In the case of sodium-ion battery cathode materials, inorganic Na_X_MO_2_ oxides, NaMPO_4_ phosphates, and NaM[M'(CN_6_)] hexacyanometalates (commonly known as Prussian blue analogs) have been tested (Xiang et al., 2015; Liu et al., 2020), and to a lesser extent, some organic MIEC polymers such as the case of Ppy (Zhou et al., 2012, Zhou et al., 2013; Zhu et al., 2013). However, in recent literature, *hOI*-MIECs started to be studied thoroughly as cathode materials for sodium-ion batteries, (e.g., Ppy/NaMnFe(CN)_6_ (Li et al., 2015), PANI/ NaNiFe(CN)_6_ (Wang Z. et al., 2017), PEDOT/ NaMnFe(CN)_6_ (Wang et al., 2020), and Ppy/NaMnO_2_ Lu et al., 2020). In the case of sodium-ion battery anode materials, the most frequent *hOI*-MIECs are based on metallic oxides such as PANI/SnO_2_ (Zhao et al., 2015) and Ppy/SnO_2_ (Yuan et al., 2018) and sulfides such as PANI/Co_3_S_4_ (Zhou et al., 2016) and Ppy/ZnS (Hou et al., 2017). It is interesting to mention that *hOI*-MIECs are also extensively used as cathodes of lithium-sulfur (Li-S) batteries such as PEDOT:PSS/S (Yang et al., 2011), Ppy/S (Han et al., 2019), and PANI/S (Wei et al., 2019). The study of MIECs as electrochemical transistors was reported long ago for typically doped Ppy (White et al., 1984), PANI (Paul et al., 1985), and PEDOT (Thackeray et al., 1985) conducting polymers, but the exploration of conducting polymers (principally PEDOT) doped with biocompatible materials such as hyaluronic acid, dextran sulfonate, heparin, pectin, guar gum, and deoxyribonucleic acid is rising fast in recent years, especially for bioelectronics purposes (Mantione et al., 2017; Tekoglu et al., 2019). In addition, a very recent report has shown that the preparation of an organic mixed-conducting particulate composite material based on PEDOT: PSS and chitosan enabled facile and effective electronic bonding between soft and rigid electronics, permitting recording of neurophysiological data at the resolution of individual neurons (Jastrzebska-Perfect et al., 2020). However, to the best of our knowledge, up to now, only carbon nanotubes (but no biocompatible inorganic nanoparticles) have been tested with organic MIECs to be evaluated for bioelectronics applications (Nie et al., 2015; Liu et al., 2019; Reddy et al., 2019; Yu et al., 2019).

## Conclusions and Perspectives

Herein, the state of the art of *hOI*-MIECs with special focus on charge carrier localization and transport at different regions including both bulk and interphase regions was discussed. In this particular case, we have mainly based our discussion by means of useful and versatile instrumental techniques such as micro-Raman and impedance spectroscopy, but other instrumental techniques can be very useful and should be considered to gain more insight into the *hOI*-MIECs transport mechanism. There is no doubt that *hOI*-MIECs have shown to be very promising for different applications, ranging from more developed applications (e.g., lithium- and sodium-ion batteries) to more emerging applications (e.g., bioelectronics), as mentioned in the previous section. However, more work is still needed to understand the charge carrier transport mechanism of such complicated systems, in order to pursue the filling of the existent gap between fundamental knowledge and applications. In our opinion, *in situ/operando* monitoring of *hOI*-MIECs during working conditions is the ideal strategy to gain more insight into this field. However, as we have discussed in this mini-review, the complexity of these particular systems (biphasic by definition and sometimes intrinsically inhomogeneous) requires the rational design of more simple devices in order to make them accessible to a broader range of *in situ* characterization experiments. We think that the oncoming focus on these experiments is crucial to shed some light on the structural and microstructural correlations of *hOI*-MIECs with the charge carrier transport mechanism.

## Author Contributions

MR, RF, and AM contributed to the conception and design of the study. DM and FP selected, compiled, and organized the literature references database. MR created the schematizations, adaptation of figure artwork, and wrote the first draft of the manuscript. DM, FP, RF, and AM wrote sections of the manuscript. All authors contributed to manuscript revision, read, and approved the submitted version.

## Conflict of Interest

The authors declare that the research was conducted in the absence of any commercial or financial relationships that could be construed as a potential conflict of interest.
